# Supplementary figures and images for: Interpretation of pre-morbid cardiac 3T MRI findings in overweight and hypertensive young adults
Source: PLoS One. 2022 Dec 1;17(12):e0278308. doi: 10.1371/journal.pone.0278308 (PMC9714856; doi:10.1371/journal.pone.0278308)

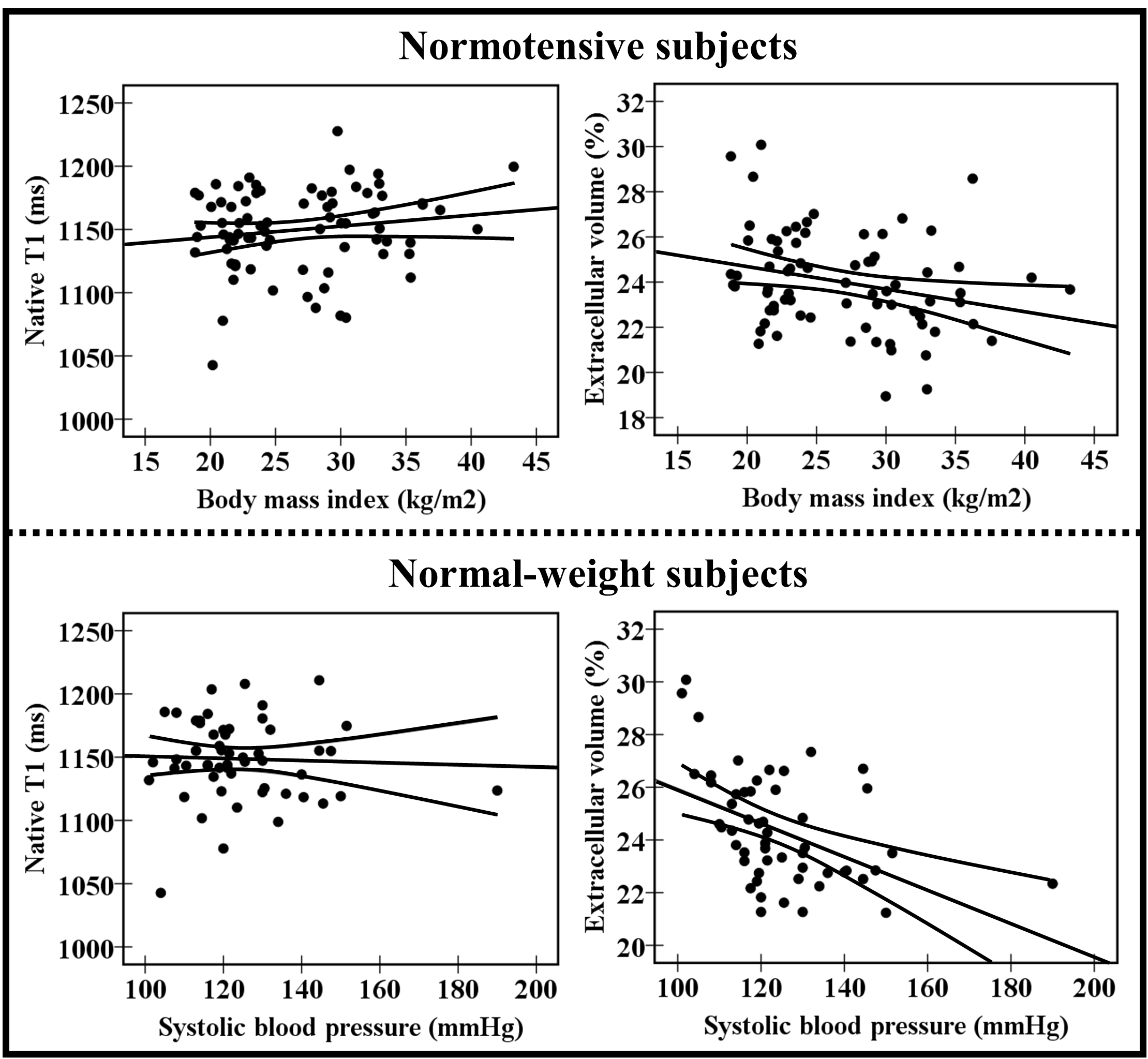

Supplement: S1 Fig — In the upper panel, only normotensive subjects are included to show the correlation between body mass index (BMI) and native T1 (P = 0.170), and between BMI and extracellular volume (ECV) (r = –0.271, P = 0.020). In the lower panel, only normal-weight subjects are included to show the correlation between systolic blood pressure (SBP) and native T1 (P = 0.751), and between SBP and ECV (r = –0.471, P < 0.001). (TIF) [file pone.0278308.s001.tif]
