# Supplementary material for: Interpretation of pre-morbid cardiac 3T MRI findings in overweight and hypertensive young adults
Source: PLoS One. 2022 Dec 1;17(12):e0278308. doi: 10.1371/journal.pone.0278308 (PMC9714856; doi:10.1371/journal.pone.0278308)
Supplement: S1 Table — (DOCX) [file pone.0278308.s002.docx]

**S1 Table. Acquisition parameters.**

| **Parameter** | **Short-axis cine** | **Native T_1_** | **Post-contrast T_1_** | **T_2_** |
| --- | --- | --- | --- | --- |
| Slice thickness (mm) | 6 | 8 | 8 | 8 |
| Interslice gap (mm) | 4 | N/A | N/A | N/A |
| Number of phases | 25 | N/A | N/A | N/A |
| Repetition time (ms) | 40.06–44.52 | 248.16–300.06 | 314.60–351.20 | 169.59–235.74 |
| Echo time (ms) | 1.19–1.31 | 1.12 | 1.01 | 1.32 |
| Flip angle (⁰) | 43–46 | 35 | 35 | 12 |
| Field of view (mm^2^) | 300-385 x 225–385 | 360 x 202–343 | 360 x  202–345 | 360 x  202–345 |
| Matrix | 256 x  192–256 | 256 x  144–218 | 192 x  108–184 | 192 x  108–186 |
| Preparation times (ms) | N/A | N/A | N/A | 0,30,55 |
