# Supplementary material for: Interpretation of pre-morbid cardiac 3T MRI findings in overweight and hypertensive young adults
Source: PLoS One. 2022 Dec 1;17(12):e0278308. doi: 10.1371/journal.pone.0278308 (PMC9714856; doi:10.1371/journal.pone.0278308)
Supplement: S2 Table — Data reported as mean ± standard deviation. *P < 0.05 versus normal-weight, †P < 0.05 versus mild overweight BMI body mass index. (DOCX) [file pone.0278308.s003.docx]

**S2 Table. Cardiac morphology and function in normotensive subjects divided on BMI.**

|  | **Normal-weight** | **Mild overweight** | **Obese** |
| --- | --- | --- | --- |
|  | **18.5–24.9 kg/m^2^** | **25**–**29.9 kg/m^2^** | **≥30 kg/m^2^** |
|  | **(*n* = 40)** | **(*n* = 15)** | **(*n* = 25)** |
| Age (years) | 34 ± 4 | 36 ± 4 | 35 ± 4 |
| Gender, male *n* (%) | 20 (50) | 7 (47) | 13 (52) |
| Body surface area (m²) | 1.9 ± 0.2 | **2.0 ± 0.1*** | **2.3 ± 0.2***,† |
| **Left ventricle** |  |  |  |
| Mass (g) | 87 ± 21 | 97 ± 19 | **108 ± 24*** |
| End-diastolic volume (ml) | 171 ± 29 | 166 ± 39 | 178 ± 34 |
| End-systolic volume (ml) | 68 ± 13 | 67 ± 24 | 72 ± 16 |
| Stroke volume (ml) | 103 ± 20 | 99 ± 18 | 105 ± 23 |
| Ejection fraction (%) | 60 ± 4 | 60 ± 5 | 59 ± 5 |
| Mass-volume ratio (g/ml) | 0.51 ± 0.09 | **0.59 ± 0.11*** | **0.62 ± 0.13*** |
| *Body surface area indexed* |  |  |  |
| Mass (g/m^2^) | 47 ± 9 | 48 ± 7 | 48 ± 9 |
| End-diastolic volume (ml/m^2^) | 92 ± 13 | 82 ± 18 | **79 ± 12*** |
| End-systolic volume (ml/m^2^) | 37 ± 7 | 33 ± 11 | **32 ± 6*** |
| Stroke volume (ml/m^2^) | 55 ± 9 | 49 ± 8 | **47 ± 9*** |
| **Right ventricle** |  |  |  |
| End-diastolic volume (ml) | 192 ± 34 | 184 ± 43 | 203 ± 41 |
| End-systolic volume (ml) | 90 ± 18 | 86 ± 27 | 98 ± 24 |
| Stroke volume (ml) | 102 ± 19 | 98 ± 18 | 105 ± 22 |
| Ejection fraction (%) | 53 ± 4 | 54 ± 5 | 52 ± 6 |
| *Body surface area indexed* |  |  |  |
| End-diastolic volume (ml/m^2^) | 103 ± 16 | **91 ± 19*** | **90 ± 14*** |
| End-systolic volume (ml/m^2^) | 48 ± 9 | 42 ± 12 | 43 ± 9 |
| Stroke volume (ml/m^2^) | 55 ± 9 | 49 ± 8 | **46 ± 8*** |

Data reported as mean ± standard deviation.
*P < 0.05 versus normal-weight, †P < 0.05 versus mild overweight
*BMI* body mass index
