# Supplementary material for: Interpretation of pre-morbid cardiac 3T MRI findings in overweight and hypertensive young adults
Source: PLoS One. 2022 Dec 1;17(12):e0278308. doi: 10.1371/journal.pone.0278308 (PMC9714856; doi:10.1371/journal.pone.0278308)
Supplement: S3 Table — Data reported as mean ± standard deviation (SD) (range for reference). Ranges for reference were calculated as mean ± t0.975,n-1 ·√((n+1)/n) · SD. *P < 0.05 versus normotensive normal-weight EDV end-diastolic volume, ESV end-systolic volume, SV stroke volume, EF ejection fraction, BSA body surface area. (DOCX) [file pone.0278308.s004.docx]

**S3 Table. Cardiac morphology and function per male subgroup.**

|  | **Normotensive** | | | | | |  | **Hypertensive** | | | | |
| --- | --- | --- | --- | --- | --- | --- | --- | --- | --- | --- | --- | --- |
|  | **Normal-weight** | | |  | **Overweight** | |  | **Normal-weight** | |  | **Overweight** | |
| **Left ventricle** |  | | | | | | | | | | | |
| Mass (g) | 104 ± 14 | (75–134) |  | **121 ± 17*** | | (85–158) |  | 122 ± 26 | (60–185) |  | **131 ± 23*** | (79–182) |
| EDV (ml) | 187 ± 28 | (126–248) |  | 186 ± 42 | | (95–277) |  | 192 ± 27 | (127–256) |  | 189 ± 36 | (108–271) |
| ESV (ml) | 74 ± 13 | (46–103) |  | 79 ± 21 | | (34–124) |  | 80 ± 14 | (47–114) |  | 76 ± 19 | (32–119) |
| SV (ml) | 112 ± 20 | (70–155) |  | 107 ± 25 | | (53–162) |  | 111 ± 17 | (70–152) |  | 114 ± 21 | (67–161) |
| EF (%) | 60 ± 5 | (50–70) |  | 58 ± 5 | | (48–68) |  | 58 ± 4 | (48–68) |  | 60 ± 5 | (49–71) |
| Mass/EDV (g/ml) | .56 ± .08 | (.39–.73) |  | **.68 ± .13*** | | (.39–.96) |  | .64 ± .08 | (.45–.82) |  | **.70 ± .09*** | (.50–.90) |
| *BSA-indexed* |  |  |  |  | |  |  |  |  |  |  |  |
| Mass (g/m^2^) | 53 ± 8 | (37–69) |  | 53 ± 6 | | (40–66) |  | 58 ± 9 | (36–81) |  | 57 ± 9 | (37–78) |
| EDV (ml/m^2^) | 95 ± 14 | (65–125) |  | **82 ± 18*** | | (44–120) |  | 92 ± 8 | (72–112) |  | 83 ± 14 | (51–114) |
| ESV (ml/m^2^) | 38 ± 7 | (23–53) |  | 35 ± 9 | | (15–54) |  | 39 ± 5 | (26–51) |  | 33 ± 8 | (15–51) |
| SV (ml/m^2^) | 57 ± 9 | (37–77) |  | **47 ± 10*** | | (25–70) |  | 53 ± 6 | (39–68) |  | 50 ± 9 | (31–69) |
| **Right ventricle** |  |  |  |  | |  |  |  |  |  |  |  |
| EDV (ml) | 212 ± 32 | (144–281) |  | 215 ± 50 | | (108–321) |  | 218 ± 40 | (124–312) |  | 215 ± 37 | (131–298) |
| ESV (ml) | 101 ± 17 | (65–138) |  | 107 ± 26 | | (51–164) |  | 108 ± 26 | (47–170) |  | 101 ± 21 | (54–147) |
| SV (ml) | 111 ± 19 | (71–151) |  | 107 ± 25 | | (53–161) |  | 110 ± 17 | (70–150) |  | 114 ± 21 | (67–160) |
| EF (%) | 52 ± 4 | (44–61) |  | 50 ± 4 | | (42–58) |  | 51 ± 4 | (40–61) |  | 53 ± 4 | (44–62) |
| *BSA-indexed* |  |  |  |  | |  |  |  |  |  |  |  |
| EDV (ml/m^2^) | 108 ± 15 | (73–143) |  | 94 ± 20 | | (51–138) |  | 104 ± 13 | (74–134) |  | 94 ± 15 | (60–128) |
| ESV (ml/m^2^) | 52 ± 9 | (32–71) |  | 47 ± 11 | | (24–70) |  | 52 ± 9 | (30–73) |  | 44 ± 9 | (25–63) |
| SV (ml/m^2^) | 56 ± 9 | (37–75) |  | **47 ± 10*** | | (25–69) |  | 53 ± 6 | (38–67) |  | 50 ± 8 | (31–69) |

Data reported as mean ± standard deviation (SD) (range for reference).
Ranges for reference were calculated as mean ± t_0.975,n-1_ ·√((n+1)/n) · SD.
*P < 0.05 versus normotensive normal-weight
*EDV* end-diastolic volume, *ESV* end-systolic volume, *SV* stroke volume, *EF* ejection fraction, *BSA* body surface area
