# Supplementary material for: Interpretation of pre-morbid cardiac 3T MRI findings in overweight and hypertensive young adults
Source: PLoS One. 2022 Dec 1;17(12):e0278308. doi: 10.1371/journal.pone.0278308 (PMC9714856; doi:10.1371/journal.pone.0278308)
Supplement: S4 Table — Data reported as mean ± standard deviation (SD) (range for reference). Ranges for reference were calculated as mean ± t0.975,n-1 ·√((n+1)/n) · SD. *P < 0.05 versus normotensive normal-weight, †P < 0.05 versus normotensive overweight EDV end-diastolic volume, ESV end-systolic volume, SV stroke volume, EF ejection fraction, BSA body surface area. (DOCX) [file pone.0278308.s005.docx]

**S4 Table. Cardiac morphology and function per female subgroup.**

|  | **Normotensive** | | | | | |  | **Hypertensive** | | | | |
| --- | --- | --- | --- | --- | --- | --- | --- | --- | --- | --- | --- | --- |
|  | **Normal weight** | | |  | **Overweight** | |  | **Normal weight** | |  | **Overweight** | |
| **Left ventricle** |  | | | | | | | | | | | |
| Mass (g) | 71 ± 12 | (45–96) |  | **86 ± 11*** | | (62–110) |  | **90 ± 12*** | (60–120) |  | **94 ± 11*** | (70–117) |
| EDV (ml) | 156 ± 21 | (112–199) |  | 161 ± 23 | | (112–209) |  | 141 ± 24 | (77–204) |  | 147 ± 27 | (88–206) |
| ESV (ml) | 62 ± 10 | (40–85) |  | 62 ± 12 | | (36–89) |  | 56 ± 8 | (34–77) |  | 56 ± 15 | (22–89) |
| SV (ml) | 93 ± 14 | (62–124) |  | 98 ± 15 | | (66–131) |  | 85 ± 18 | (38–133) |  | 92 ± 14 | (61–122) |
| EF (%) | 60 ± 4 | (51–69) |  | 61 ± 5 | | (50–72) |  | 60 ± 4 | (50–71) |  | 63 ± 4 | (52–73) |
| Mass/EDV (g/ml) | .46 ± .06 | (.34–.57) |  | **.54 ± .06*** | | (.41–.67) |  | **.66 ± .15*** | (.25–1.06) |  | **.65 ± .09***,† | (.44–.86) |
| *BSA-indexed* |  |  |  |  | |  |  |  |  |  |  |  |
| Mass (g/m^2^) | 40 ± 6 | (27–53) |  | 42 ± 4 | | (32–51) |  | **50 ± 6*** | (34–66) |  | **46 ± 4*** | (36–56) |
| EDV (ml/m^2^) | 89 ± 12 | (63–114) |  | **78 ± 9*** | | (58–98) |  | 78 ± 12 | (47–110) |  | **72 ± 13*** | (44–101) |
| ESV (ml/m^2^) | 36 ± 6 | (23–48) |  | **30 ± 6*** | | (18–43) |  | 31 ± 4 | (20–42) |  | **27 ± 7*** | (12–43) |
| SV (ml/m^2^) | 53 ± 8 | (36–70) |  | 48 ± 6 | | (35–60) |  | 47 ± 9 | (24–71) |  | **45 ± 7*** | (30–61) |
| **Right ventricle** |  |  |  |  | |  |  |  |  |  |  |  |
| EDV (ml) | 172 ± 22 | (124–219) |  | 177 ± 22 | | (130–224) |  | 152 ± 23 | (91–213) |  | 159 ± 28 | (97–222) |
| ESV (ml) | 78 ± 12 | (53–104) |  | 80 ± 15 | | (48–111) |  | 66 ± 10 | (39–93) |  | 69 ± 17 | (30–107) |
| SV (ml) | 93 ± 15 | (61–125) |  | 97 ± 15 | | (66–129) |  | 86 ± 18 | (39–134) |  | 91 ± 13 | (61–120) |
| EF (%) | 54 ± 5 | (45–64) |  | 55 ± 6 | | (43–67) |  | 56 ± 6 | (41–72) |  | 57 ± 5 | (47–67) |
| *BSA-indexed* |  |  |  |  | |  |  |  |  |  |  |  |
| EDV (ml/m^2^) | 98 ± 13 | (70–126) |  | **86 ± 10*** | | (65–107) |  | 85 ± 10 | (58–112) |  | **79 ± 14*** | (48–109) |
| ESV (ml/m^2^) | 45 ± 7 | (30–60) |  | 39 ± 8 | | (22–55) |  | 37 ± 5 | (23–50) |  | **34 ± 8*** | (16–52) |
| SV (ml/m^2^) | 53 ± 8 | (35–71) |  | 47 ± 6 | | (35–60) |  | 48 ± 9 | (25–71) |  | **45 ± 7*** | (30–60) |

Data reported as mean ± standard deviation (SD) (range for reference).
Ranges for reference were calculated as mean ± t_0.975,n-1_ ·√((n+1)/n) · SD.
*P < 0.05 versus normotensive normal-weight, †P < 0.05 versus normotensive overweight
*EDV* end-diastolic volume, *ESV* end-systolic volume, *SV* stroke volume, *EF* ejection fraction, *BSA* body surface area
