# Supplementary material for: Interpretation of pre-morbid cardiac 3T MRI findings in overweight and hypertensive young adults
Source: PLoS One. 2022 Dec 1;17(12):e0278308. doi: 10.1371/journal.pone.0278308 (PMC9714856; doi:10.1371/journal.pone.0278308)
Supplement: S5 Table — Data reported as mean ± standard deviation. *P < 0.05 versus normal-weight, †P < 0.05 versus mild overweight BMI body mass index. (DOCX) [file pone.0278308.s006.docx]

**S5 Table. Cardiac morphology and function in normotensive males divided on BMI.**

|  | **Normal-weight** | **Mild overweight** | **Obese** |
| --- | --- | --- | --- |
|  | **18.5–24.9 kg/m^2^** | **25**–**29.9 kg/m^2^** | **≥30 kg/m^2^** |
|  | **(*n* = 20)** | **(*n* = 7)** | **(*n* = 13)** |
| Age (years) | 33 ± 3 | 34 ± 4 | 35 ± 4 |
| Body surface area (m²) | 2.0 ± 0.1 | 2.1 ± 0.1 | **2.4 ± 0.2***,† |
| **Left ventricle** |  |  |  |
| Mass (g) | 104 ± 14 | 112 ± 15 | **126 ± 16*** |
| End-diastolic volume (ml) | 187 ± 28 | 181 ± 50 | 189 ± 39 |
| End-systolic volume (ml) | 74 ± 13 | 77 ± 29 | 80 ± 16 |
| Stroke volume (ml) | 112 ± 20 | 104 ± 24 | 109 ± 27 |
| Ejection fraction (%) | 60 ± 5 | 58 ± 6 | 58 ± 4 |
| Mass-volume ratio (g/ml) | 0.56 ± 0.08 | 0.65 ± 0.14 | **0.69 ± 0.14*** |
| *Body surface area indexed* |  |  |  |
| Mass (g/m^2^) | 53 ± 8 | 53 ± 4 | 54 ± 7 |
| End-diastolic volume (ml/m^2^) | 95 ± 14 | 86 ± 23 | **80 ± 15*** |
| End-systolic volume (ml/m^2^) | 38 ± 7 | 37 ± 14 | 34 ± 6 |
| Stroke volume (ml/m^2^) | 57 ± 9 | 49 ± 11 | **46 ± 10*** |
| **Right ventricle** |  |  |  |
| End-diastolic volume (ml) | 212 ± 32 | 200 ± 56 | 223 ± 46 |
| End-systolic volume (ml) | 101 ± 17 | 97 ± 33 | 113 ± 21 |
| Stroke volume (ml) | 111 ± 19 | 103 ± 24 | 110 ± 26 |
| Ejection fraction (%) | 52 ± 4 | 52 ± 4 | 49 ± 3 |
| *Body surface area indexed* |  |  |  |
| End-diastolic volume (ml/m^2^) | 108 ± 16 | 95 ± 26 | 94 ± 17 |
| End-systolic volume (ml/m^2^) | 52 ± 9 | 46 ± 15 | 48 ± 8 |
| Stroke volume (ml/m^2^) | 56 ± 9 | 49 ± 11 | **46 ± 10*** |

Data reported as mean ± standard deviation.
*P < 0.05 versus normal-weight, †P < 0.05 versus mild overweight
*BMI* body mass index
