# Supplementary material for: Interpretation of pre-morbid cardiac 3T MRI findings in overweight and hypertensive young adults
Source: PLoS One. 2022 Dec 1;17(12):e0278308. doi: 10.1371/journal.pone.0278308 (PMC9714856; doi:10.1371/journal.pone.0278308)
Supplement: S6 Table — Data reported as mean ± standard deviation. *P < 0.05 versus normal-weight, †P < 0.05 versus mild overweight BMI body mass index. (DOCX) [file pone.0278308.s007.docx]

**S6 Table. Cardiac morphology and function in normotensive females divided on BMI.**

|  | **Normal-weight** | **Mild overweight** | **Obese** |
| --- | --- | --- | --- |
|  | **18.5–24.9 kg/m^2^** | **25**–**29.9 kg/m^2^** | **≥30 kg/m^2^** |
|  | **(*n* = 20)** | **(*n* = 8)** | **(*n* = 12)** |
| Age (years) | 34 ± 4 | 37 ± 3 | 35 ± 5 |
| Body surface area (m²) | 1.8 ± 0.1 | **2.0 ± 0.1*** | **2.1 ± 0.1***,† |
| **Left ventricle** |  |  |  |
| Mass (g) | 71 ± 12 | **83 ± 11*** | **88 ± 11*** |
| End-diastolic volume (ml) | 156 ± 21 | 153 ± 22 | 166 ± 22 |
| End-systolic volume (ml) | 62 ± 10 | 59 ± 14 | 65 ± 11 |
| Stroke volume (ml) | 93 ± 14 | 95 ± 11 | 101 ± 17 |
| Ejection fraction (%) | 60 ± 4 | 62 ± 4 | 61 ± 6 |
| Mass-volume ratio (g/ml) | 0.46 ± 0.06 | **0.55 ± 0.05*** | **0.53 ± 0.07*** |
| *Body surface area indexed* |  |  |  |
| Mass (g/m^2^) | 40 ± 6 | 43 ± 5 | 41 ± 4 |
| End-diastolic volume (ml/m^2^) | 89 ± 12 | 78 ± 11 | **78 ± 8*** |
| End-systolic volume (ml/m^2^) | 36 ± 6 | 30 ± 7 | 30 ± 5 |
| Stroke volume (ml/m^2^) | 53 ± 8 | 48 ± 5 | 47 ± 7 |
| **Right ventricle** |  |  |  |
| End-diastolic volume (ml) | 172 ± 22 | 171 ± 25 | 181 ± 20 |
| End-systolic volume (ml) | 78 ± 12 | 77 ± 16 | 82 ± 14 |
| Stroke volume (ml) | 93 ± 15 | 95 ± 11 | 99 ± 17 |
| Ejection fraction (%) | 54 ± 5 | 56 ± 4 | 55 ± 7 |
| *Body surface area indexed* |  |  |  |
| End-diastolic volume (ml/m^2^) | 98 ± 13 | 87 ± 12 | **85 ± 9*** |
| End-systolic volume (ml/m^2^) | 45 ± 7 | 39 ± 8 | 38 ± 7 |
| Stroke volume (ml/m^2^) | 53 ± 8 | 48 ± 5 | 46 ± 7 |

Data reported as mean ± standard deviation.
*P < 0.05 versus normal-weight, †P < 0.05 versus mild overweight
*BMI* body mass index
