# Supplementary material for: Interpretation of pre-morbid cardiac 3T MRI findings in overweight and hypertensive young adults
Source: PLoS One. 2022 Dec 1;17(12):e0278308. doi: 10.1371/journal.pone.0278308 (PMC9714856; doi:10.1371/journal.pone.0278308)
Supplement: S7 Table — Data reported as mean ± standard deviation. *P < 0.05 versus normal-weight, †P < 0.05 versus mild overweight BMI body mass index. (DOCX) [file pone.0278308.s008.docx]

**S7 Table. Cardiac morphology and function in hypertensive subjects divided on BMI.**

|  | **Normal-weight** | **Mild overweight** | **Obese** |
| --- | --- | --- | --- |
|  | **18.5–24.9 kg/m^2^** | **25**–**29.9 kg/m^2^** | **≥30 kg/m^2^** |
|  | **(*n* = 17)** | **(*n* = 14)** | **(*n* = 15)** |
| Age (years) | 36 ± 3 | 36 ± 4 | 36 ± 4 |
| Gender, male *n* (%) | 10 (59) | 8 (57) | 6 (40) |
| Body surface area (m²) | 2.0 ± 0.2 | 2.1 ± 0.2 | **2.2 ± 0.2*** |
| **Left ventricle** |  |  |  |
| Mass (g) | 109 ± 27 | 111 ± 28 | 113 ± 24 |
| End-diastolic volume (ml) | 171 ± 36 | 173 ± 42 | 163 ± 35 |
| End-systolic volume (ml) | 70 ± 17 | 67 ± 23 | 63 ± 17 |
| Stroke volume (ml) | 100 ± 21 | 106 ± 21 | 99 ± 20 |
| Ejection fraction (%) | 59 ± 4 | 62 ± 5 | 61 ± 4 |
| Mass-volume ratio (g/ml) | 0.64 ± 0.11 | 0.64 ± 0.09 | 0.70 ± 0.09 |
| *Body surface area indexed* |  |  |  |
| Mass (g/m^2^) | 55 ± 9 | 52 ± 9 | 51 ± 9 |
| End-diastolic volume (ml/m^2^) | 86 ± 12 | 81 ± 14 | **74 ± 14*** |
| End-systolic volume (ml/m^2^) | 35 ± 6 | 32 ± 9 | **29 ± 7*** |
| Stroke volume (ml/m^2^) | 51 ± 8 | 50 ± 7 | 45 ± 9 |
| *Height^2.7^ indexed* |  |  |  |
| Mass (g/m^2.7^) | 22 ± 3 | 22 ± 3 | 25 ± 5 |
| End-diastolic volume (ml/m^2.7^) | 34 ± 4 | 35 ± 5 | 36 ± 8 |
| End-systolic volume (ml/m^2.7^) | 14 ± 2 | 14 ± 3 | 14 ± 4 |
| Stroke volume (ml/m^2.7^) | 20 ± 3 | 22 ± 3 | 22 ± 5 |
| **Right ventricle** |  |  |  |
| End-diastolic volume (ml) | 191 ± 47 | 195 ± 41 | 177 ± 44 |
| End-systolic volume (ml) | 91 ± 30 | 90 ± 23 | 79 ± 26 |
| Stroke volume (ml) | 100 ± 21 | 105 ± 21 | 99 ± 21 |
| Ejection fraction (%) | 53 ± 6 | 54 ± 4 | 56 ± 5 |
| *Body surface area indexed* |  |  |  |
| End-diastolic volume (ml/m^2^) | 96 ± 15 | 92 ± 13 | **81 ± 18*** |
| End-systolic volume (ml/m^2^) | 46 ± 11 | 42 ± 8 | **36 ± 10*** |
| Stroke volume (ml/m^2^) | 51 ± 7 | 50 ± 7 | 45 ± 9 |
| *Height^2.7^ indexed* |  |  |  |
| End-diastolic volume (ml/m^2.7^) | 38 ± 5 | 40 ± 4 | 39 ± 9 |
| End-systolic volume (ml/m^2.7^) | 18 ± 3 | 18 ± 3 | 17 ± 5 |
| Stroke volume (ml/m^2.7^) | 20 ± 3 | 22 ± 3 | 22 ± 4 |

Data reported as mean ± standard deviation.
*P < 0.05 versus normal-weight, †P < 0.05 versus mild overweight.
*BMI* body mass index
