# Supplementary material for: Interpretation of pre-morbid cardiac 3T MRI findings in overweight and hypertensive young adults
Source: PLoS One. 2022 Dec 1;17(12):e0278308. doi: 10.1371/journal.pone.0278308 (PMC9714856; doi:10.1371/journal.pone.0278308)
Supplement: S8 Table — Data reported as mean ± standard deviation. *P < 0.05 versus normotensive normal-weight. (DOCX) [file pone.0278308.s009.docx]

**S8 Table. Indexation of volumes and mass for different body scales.**

|  | **Normotensive** | |  | **Hypertensive** | |
| --- | --- | --- | --- | --- | --- |
|  | **Normal-weight** | **Overweight** |  | **Normal-weight** | **Overweight** |
| **Indexed for body surface area** | | | | | |
| **Left ventricle** |  |  |  |  |  |
| Mass (g/m^2^) | 47 ± 9 | 48 ± 8 |  | **55 ± 9*** | 52 ± 9 |
| End-diastolic volume (ml/m^2^) | 92 ± 13 | **80 ± 14*** |  | 86 ± 12 | **78 ± 14*** |
| Stroke volume (ml/m^2^) | 55 ± 9 | **47 ± 8*** |  | 51 ± 8 | **47 ± 8*** |
| **Right ventricle** |  |  |  |  |  |
| End-diastolic volume (ml/m^2^) | 103 ± 15 | **90 ± 16*** |  | 96 ± 15 | **86 ± 16*** |
| **Indexed for lean body mass** | | | | | |
| **Left ventricle** |  |  |  |  |  |
| Mass (g/kg) | 1.8 ± 0.3 | 1.7 ± 0.2 |  | **2.1 ± 0.3*** | 1.9 ± 0.2 |
| End-diastolic volume (ml/kg) | 3.5 ± 0.6 | **2.9 ± 0.6*** |  | 3.2 ± 0.4 | **2.8 ± 0.5*** |
| Stroke volume (ml/kg) | 2.1 ± 0.4 | **1.7 ± 0.4*** |  | 1.9 ± 0.3 | **1.7 ± 0.3*** |
| **Right ventricle** |  |  |  |  |  |
| End-diastolic volume (ml/kg) | 4.0 ± 0.6 | **3.2 ± 0.6*** |  | 3.6 ± 0.4 | **3.1 ± 0.5*** |
| **Indexed for height** | | | | | |
| **Left ventricle** |  |  |  |  |  |
| Mass (g/m) | 49 ± 11 | **58 ± 11*** |  | **60 ± 12*** | **63 ± 12*** |
| End-diastolic volume (ml/m) | 96 ± 14 | 98 ± 18 |  | 94 ± 16 | 94 ± 19 |
| Stroke volume (ml/m) | 58 ± 10 | 58 ± 11 |  | 55 ± 10 | 57 ± 10 |
| **Right ventricle** |  |  |  |  |  |
| End-diastolic volume (ml/m) | 108 ± 16 | 110 ± 21 |  | 104 ± 20 | 104 ± 21 |
| **Indexed for height^1.7^** | | | | | |
| **Left ventricle** |  |  |  |  |  |
| Mass (g/m^1.7^) | 33 ± 7 | **39 ± 7*** |  | **39 ± 7*** | **42 ± 8*** |
| End-diastolic volume (ml/m^1.7^) | 64 ± 9 | 66 ± 11 |  | 62 ± 9 | 63 ± 12 |
| Stroke volume (ml/m^1.7^) | 38 ± 6 | 39 ± 7 |  | 36 ± 6 | 38 ± 6 |
| **Right ventricle** |  |  |  |  |  |
| End-diastolic volume (ml/m^1.7^) | 72 ± 10 | 74 ± 13 |  | 69 ± 11 | 70 ± 13 |
| **Indexed for height^2.7^** | | | | | |
| **Left ventricle** |  |  |  |  |  |
| Mass (g/m^2.7^) | 18 ± 4 | **22 ± 3*** |  | **22 ± 3*** | **24 ± 4*** |
| End-diastolic volume (ml/m^2.7^) | 36 ± 5 | 37 ± 6 |  | 34 ± 4 | 35 ± 6 |
| Stroke volume (ml/m^2.7^) | 22 ± 3 | 22 ± 4 |  | 20 ± 3 | 22 ± 4 |
| **Right ventricle** |  |  |  |  |  |
| End-diastolic volume (ml/m^2.7^) | 40 ± 6 | 42 ±7 |  | 38 ± 5 | 39 ± 7 |

Data reported as mean ± standard deviation.

*P < 0.05 versus normotensive normal-weight
