# Supplementary material for: Interpretation of pre-morbid cardiac 3T MRI findings in overweight and hypertensive young adults
Source: PLoS One. 2022 Dec 1;17(12):e0278308. doi: 10.1371/journal.pone.0278308 (PMC9714856; doi:10.1371/journal.pone.0278308)
Supplement: S9 Table — Data reported as mean ± standard deviation. Indexed indexed with body surface area, EDV end-diastolic volume, ESV end-systolic volume, SV stroke volume. (DOCX) [file pone.0278308.s010.docx]

**S9 Table. Differences between hypertensive subjects on presence anti-hypertensive medication.**

|  | **Normal-weight** | | |  | **Overweight** | | |
| --- | --- | --- | --- | --- | --- | --- | --- |
|  | **Medication** | |  |  | **Medication** | |  |
|  | **Yes**  **(n = 10)** | **No**  **(n = 7)** | **P *P*** |  | **Yes (n = 17)** | **No (n = 13)** | **P** |
| Age (years) | 38 ± 2 | 35 ± 4 | 0.078 |  | 36 ± 4 | 35 ± 4 | 0.334 |
| Gender, male *n* (%) | 4 (40) | 6 (86) | 0.065 |  | 7 (41) | 7 (58) | 0.381 |
| Body surface area (m²) | 1.9 ± 0.2 | 2.0 ± 0.1 | 0.379 |  | 2.1 ± 0.2 | 2.2 ± 0.2 | 0.815 |
| **Left ventricle** |  |  |  |  |  |  |  |
| Indexed mass (g/m^2^) | 52 ± 9 | 59 ± 8 | 0.105 |  | 51 ± 9 | 52 ± 10 | 0.717 |
| Indexed EDV (ml/m^2^) | 82 ± 12 | 92 ± 10 | 0.128 |  | 78 ± 17 | 76 ± 11 | 0.702 |
| Indexed ESV (ml/m^2^) | 33 ± 5 | 39 ± 6 | 0.055 |  | 31 ± 8 | 29 ± 7 | 0.595 |
| Indexed SV (ml/m^2^) | 50 ± 9 | 53 ± 7 | 0.415 |  | 48 ± 9 | 47 ± 6 | 0.868 |
| **Right ventricle** |  |  |  |  |  |  |  |
| Indexed EDV (ml/m^2^) | 92 ± 13 | 103 ± 17 | 0.157 |  | 84 ± 17 | 89 ± 15 | 0.465 |
| Indexed ESV (ml/m^2^) | 42 ± 9 | 50 ± 12 | 0.144 |  | 37 ± 10 | 41 ± 9 | 0.237 |
| Indexed SV (ml/m^2^) | 49 ± 8 | 52 ± 7 | 0.441 |  | 47 ± 9 | 47 ± 7 | 0.960 |
| **Global mapping results** |  |  |  |  |  |  |  |
| Native T_1_ (ms) | 1148 ± 37 | 1156 ± 35 | 0.685 |  | 1157 ± 32 | 1147 ± 44 | 0.494 |
| Extracellular volume (%) | 24.0 ± 2.0 | 23.3 ± 1.8 | 0.466 |  | 23.7 ± 2.6 | 23.2 ± 2.6 | 0.609 |
| T_2_ (ms) | 38.1 ± 1.7 | 38.3 ± 0.9 | 0.837 |  | 37.6 ± 2.3 | 37.3 ± 2.1 | 0.728 |

Data reported as mean ± standard deviation. *Indexed* indexed with body surface area, *EDV* end-diastolic volume, *ESV* end-systolic volume, *SV* stroke volume
