# Supplementary material for: Interpretation of pre-morbid cardiac 3T MRI findings in overweight and hypertensive young adults
Source: PLoS One. 2022 Dec 1;17(12):e0278308. doi: 10.1371/journal.pone.0278308 (PMC9714856; doi:10.1371/journal.pone.0278308)
Supplement: Attachment — Submitted filename: PONE-D-21-38012 - Rebuttal letter.docx [file pone.0278308.s011.docx]

Dear editor and reviewer,

Thank you for handling our manuscript and for providing helpful comments.
Please find below our point-to-point response to each comment.
As requested, we uploaded a marked-up copy of the manuscript with changes tracked and an unmarked version without changes tracked.
Also, we uploaded the newest versions of the supplementary materials.

Your sincerely,

Gert Jan Snel – Corresponding author

**Major comments:
1. Obese participants are only reported separately for some comparisons but are combined with the overweight group in most comparisons. What is the rationale for this limited analysis and can the authors differentiate between obese and overweight participants in additional studies? The majority of overweight normotensives are obese, what was the number of obese vs. overweight hypertensive.**

We thank the reviewer for these questions. One of our hypotheses was that being overweight without hypertension would result in measurably larger cardiac volumes compared to being normal weight. The cardiac adaptation in our normotensive overweight population was non-significantly different from controls. We therefore performed an analysis in which we subdivided our normotensive overweight group into mild overweight and obese.

We agree that it would also be of interest to subdivide the hypertensive overweight population (n = 29) into mild overweight (n = 14) and obese (n = 15). We compared these new subgroups to our hypertensive normal-weight group and investigated the impact of overweight in these subjects. The differences between these groups were all non-significant and are added in supplementary tables (S14 and S15). The following text is added to the section Results – Cardiac morphology and function:

*“**Results of cardiac morphology and function in hypertensive populations with normal-weight, mild overweight and obese are reported in S14 and S15 Tables. Although not significant, in the obese group, LVM was higher and cardiac volumes were lower. After height^2.7^ indexation, the mild overweight and obese groups showed non-significantly higher end-diastolic volumes, stroke volumes and LVM compared to normal-weights.”*


**2. The authors discuss results from obese males. Are these results reported in a Table? Please indicate the number of obese females is not indicated. the number of obese males and females.**

Thank you for this clarification request. Results from obese males were indeed not reported in a table. We added supplementary tables to report the results of both obese males (n = 13, Tables S10 and S11) and obese females (n = 12, Tables S12 and S13). We referred to these tables in the section Results – Cardiac morphology and function.

For clarity, we rephrased the sentence of the discussion to state:

*“**Our findings in overweight groups were consistent with previous studies reporting increasing LVM with higher BMI versus controls; in mature adults (aged 61 ± 9 years, no sex-specific results) [10–12,29], in young adult obese males (aged 30 ± 7 years, BMI 35 ± 3 kg/m^2^) [30], and in severely obese adolescents (aged 18 ± 4 years, BMI 41 ± 6 kg/m^2^) [31].”*


**3. Table S4 shows that BSA masks LV mass but accentuates differences in volumes (ESV, EDV, SV), particularly in obese patients and to a lesser extent RV volumes (EDV; SV in obese). In Table S6 male normotensives overall follow this pattern in overweight normotensives but Table S8 shows that in females, BSA masks mass in normotensive but not in hypertensives and volumes are accentuated in both overweight groups. Please elaborate**

In both normotensive overweight and hypertensive overweight groups, BSA-indexation indeed resulted in lower volumes compared to normal-weights. This pattern was observed in the entire population (Table S4 and S14), and separately for males (Table S6) and females (Table S8).
BSA-indexation masked LV mass in normotensive overweights as well. There was less masking of LV mass in hypertensive overweights because of the additional cardiac adaptation to hypertension. As a result, BSA-indexed LV mass remained higher in both hypertensive overweight males (Table S6) and females (Table S8) compared to controls, though only significant in females.

We added the following clarifying sentence in Results – Indexation Methods:

*“**In the hypertensive overweight group, BSA-indexed LVM remained higher compared to controls, though only significant in the female subgroup (S6 and S8 Tables).”*

We also rephrased one sentence in the abstract to state:

*“Body surface area (BSA) indexation resulted in LVM normalization in overweights (48 ± 8 g/m^2^) versus controls (47 ± 9 g/m^2^), but not in hypertensives (55 ± 9 g/m^2^) and hypertensive overweights (52 ± 9 g/m^2^).”*


**4. 4. In Table 2, differences in the global mapping the values extracellular volume and T2 are virtually identical in overweight normotensive and both hypertensive groups, although, some are significant. What is the relevance of these differences?**

Thank you for pointing this out. We originally rounded T_2_ and extracellular volume (ECV) up to the closest integer, which made these results appear more identical and statistically relevant differences less apparent. We added one decimal for T_2_ and ECV in Tables 2 and S18.

T_2_ mapping values were only significantly different in the hypertensive overweight group compared to controls, because of higher heart rates causing lower T_2_ values, which is a known effect in SSFP-based sequences, as stated in the discussion (R256-258).

All risk groups showed lower ECV values compared to controls, though not significant in the normal-weight hypertensive group. These changes suggest that the increased LV mass mainly related to hypertrophy of myocytes, and to a lesser extent fibrosis (since T_1_ values were similar). We rephrased one sentence in the discussion to state:

*“Our results suggest that increased LVM in young adults with overweight and/or hypertension is mainly caused by increased myocyte mass and to a lesser extent by fibrosis, as evidenced by similar T_1_ values and lower ECV.”*


**Minor comments:
1. Please define the parameters for obese in the Methods section**

Thank you for this request. In the inclusion criteria of the study, we used a BMI of 25 kg/m^2^ as cut-off to classify subjects as either normal-weight or overweight. Since we considered the severity of overweight to be potentially of interest during the analysis, we subdivided the overweight population into mild overweight and obese to explore possible differences. Therefore, we defined mild overweight and obese in the section Methods – Statistical Analysis by adding the following text:

*“For some analyses, overweight subjects were further subdivided into mild overweight (BMI 25–29.9 kg/m^2^) and obese (BMI ≥ 30 kg/m^2^) to further explore possible differences in cardiac MRI parameters.”*

In the section Results – Cardiac morphology and function, we removed “BMI 25-29.9 kg/m^2^” and “BMI 30-43 kg/m^2^”, to avoid redundancy after defining these groups in the Methods section. Furthermore, we renamed overweight (BMI 25–29.9 kg/m^2^) as mild overweight to prevent confusion with the original overweight group as stated in inclusion criteria (BMI ≥ 25 kg/m^2^).


**2. In Table S8 it states that “data reported as mean ± SD”. Please similarly indicate, either in the Methods section or in the legend for each table.**

In the original manuscript, we stated in the section Methods – Statistical analysis that “Continuous variables were reported as mean ± standard deviation”. We agree that it would be clearer to also include this in the table legends. We added this information in all relevant tables; 1, 2, S4, S16 and S18.
