# Supplementary material for: Interpretation of pre-morbid cardiac 3T MRI findings in overweight and hypertensive young adults
Source: PLoS One. 2022 Dec 1;17(12):e0278308. doi: 10.1371/journal.pone.0278308 (PMC9714856; doi:10.1371/journal.pone.0278308)
Supplement: Attachment — Submitted filename: PONE-D-21-38012R1 - Rebuttal letter.docx [file pone.0278308.s012.docx]

Dear editor and reviewers,

Thank you for handling our manuscript and providing helpful comments.
Please find below our point-to-point response to each comment.
As requested, we uploaded a marked-up copy of the manuscript with tracked changes and an unmarked version.
Also, we uploaded the newest versions of the supplementary materials.

Your sincerely,

Gert Jan Snel – Corresponding author

**Reviewer #1: (No Response)**

**Reviewer #2:** 

**Abstract
1. Please state explicitly the aims of your work in the abstract within the first sentences.**

Thank you for this clarification request. We rephrased the first sentences in the abstract to state:

*“In young adults, overweight and hypertension possibly already trigger cardiac remodeling as seen in mature adults, potentially overlapping non-ischemic cardiomyopathy findings.* *To this end, in young overweight and hypertensive adults, we aimed to investigate changes in left ventricular mass (LVM) and cardiac volumes, and the impact of different body scales for indexation. We also aimed to explore the presence of myocardial fibrosis, fat and edema, and changes in cellular mass with extracellular volume (ECV), T_1_ and T_2_ tissue characteristics.”*

**Introduction
2. Page 3 lines 63-64: perhaps the word “volume” is missing in “cardiac volumes caused by continuous high blood circulation”.**

Thank you for pointing this out. We added the word “volume” in that sentence to state:

*“Large cohort studies in adults over 40 years of age demonstrated that increased BMI is associated with increased LVM and cardiac volumes caused by continuous high blood volume circulation, while hypertension is associated with increased LVM caused by elevated systemic vascular resistance [10–12].”*

**Materials and methods
3. Please report a sample size calculation if such analysis was performed in the study planning stages. Otherwise, I would suggest conducting a statistical power analysis, given the high number of comparisons performed and the relatively small sizes of the study groups.**

Thank you for this request. We indeed performed a sample size analysis in the study planning stages. We added the following paragraph to the “Statistical Analysis” section:

*“**The sample size was calculated using previously published data in athletes [29]. In that study, the mean difference between athletes and controls was ±9 g, and the standard deviation was ±13 g. With a one-sided significance level of 0.05 (i.e., α) and 80% power (i.e., β=0.20), a sample size of 26 was needed per group.”*

Our group sizes of controls, normotensive overweight and hypertensive overweight were sufficiently large. Due to the relatively low prevalence of hypertension in the general normal-weight population between 18 and 45 years of age, our included hypertensive normal-weight group size was smaller than the number calculated in our power analysis. We added the smaller group size as a limitation by stating:

*“**Fourth, the hypertensive normal-weight group was smaller than the sample size calculated with the power analysis.”*

**Results
4. While I appreciate the detailed report on all the analysis performed, I found very dispersive to scroll between 18 different supplementary tables. Therefore, I would suggest the authors to reduce such number, perhaps by collapsing similar tables into one or by avoiding to report non-fundamental data.**

We thank the reviewer for this suggestion. We removed the supplementary tables containing solely P-values since the fundamental data and significant differences are reported in other tables. As a result, the number of supplementary tables was reduced from 18 to 9.
